# Eosinophilia and risk of incident end stage kidney disease

**DOI:** 10.1186/s12882-020-1685-3

**Published:** 2020-01-13

**Authors:** Anam Tariq, Keisuke Okamato, Azka Tariq, Avi Z. Rosenberg, Karim M. Soliman, David W. Ploth, Mohamed G. Atta, Blaithin A. McMahon

**Affiliations:** 1 Division of Nephrology, Johns Hopkins University, 1830 Monument Street, Suite 416, Baltimore, Maryland 21287 USA; 2 Division of Nephrology, Medical University of South Carolina, Charleston, SC USA; 3 Division of Pathology, Johns Hopkins University, Baltimore, MD USA

**Keywords:** Eosinophilia, Kidney biopsy, Interstitial nephritis, End-stage-kidney-disease, Inflammation

## Abstract

**Background:**

Eosinophils in kidney disease are poorly understood and are often incidental findings on kidney biopsy. Eosinophilia in blood and renal biopsy tissue is associated with a host of immune and non-immune kidney diseases. The significance of eosinophilia in renal diseases has not been well addressed. We evaluated the presence of peripheral eosinophilia (> 4% of blood leukocytes) with biopsy tissue eosinophilia and their association with end-stage-kidney-disease (ESKD).

**Methods:**

A nested case-control (2:1) of patients who underwent kidney biopsies at Johns Hopkins Hospital and Medical University of South Carolina from 2004 to 2018 were included in the study. From the 616 eligible patients, 178 patients were identified through the registry of kidney biopsies as 18 years or older without missing biopsy reports or hematology results. Controls (*n* = 154) had no ESKD at the time of case (*n* = 24) designation and were assembled using incident density sampling and matched on age and sex. The association of peripheral eosinophilia (> 4% of peripheral blood leukocytes) with the risk of progression to ESKD was evaluated using conditional logistic model after adjusting for clinical demographics.

**Results:**

Among 178 patients, 65 (37%) had peripheral eosinophilia and 113 (63%) had no eosinophilia. Compared to patients without eosinophilia, patients with peripheral eosinophilia were notably male and had a higher serum creatinine at the time of their biopsy. Peripheral eosinophilia was associated with higher risk of ESKD (OR 15.9 [1.9, 134.7]) adjusted for patient demographics including hypertension, proteinuria and eGFR at the time of kidney biopsy. Peripheral eosinophilia had a significant linear association with kidney tissue eosinophils, 22 (standard deviation [SD] 20) per high power field (hpf) in 4–10% peripheral eosinophilia, 19 (SD 18) per hpf in ≥10% eosinophilia and 3 (SD 7) per hpf in no eosinophilia (*P* <  0.001).

**Conclusions:**

Peripheral eosinophilia is an independent predictor of tissue eosinophilia and subsequent progression to ESKD. Peripheral eosinophilia may be an early biomarker for underlying inflammation and disease, but further studies to investigate this clinical association are warranted.

## Background

Production of eosinophils is closely related with inflammation and the immune response to parasitic illness, asthma, hypersensitivity reactions and allergic responses [1, 2]. Eosinophil elevation has been hypothesized to cause tissue and organ damage by cytotoxic effects from reactive oxygen species, and other proteins [3]. The role of hypereosinophilia in kidney failure has been reported in a small number of case-reports and observational studies [1, 2]. The case-reports highlight the presence of peripheral eosinophilia in interstitial nephritis (IN) secondary to medications and rare autoimmune diseases, such as IgG4-related kidney disease and antineutrophil cytoplasmic antibody (ANCA)-associated vasculitis, which comprises of granulomatosis with polyangiitis (GPA, previously known as Wegener’s granulomatosis), microscopic polyangiitis (MPA) and eosinophilic granulomatosis with polyangiitis (EGPA, previously known as Churg-Strauss syndrome) [4–9]. Other rare, but distinctive diseases include neuromyelitis optica, bullous pemphigoid, autoimmune myocarditis, HIV, and Hyperimmunoglobulin E Syndrome [7, 10, 11]. We conducted this study to address the role of peripheral eosinophilia and progression to ESKD. We hypothesized that peripheral eosinophilia correlates with higher tissue eosinophilia and both are independently associated with the increased risk of progression to ESKD.

## Methods

Our case-control study was nested within a longitudinal prospective study of patients who underwent native or transplant kidney biopsies at Johns Hopkins Hospital from 2004 to 2018 and at Medical University of South Carolina from 2017 to 2018. Eligible cases (*n* = 24) included those with confirmed diagnosis of incident ESKD after enrollment. Controls (*n* = 154) consisted of patients who underwent kidney biopsy from 2004 to 2018 who did not progress to ESKD after enrollment. This study was approved by the institutional review boards at Johns Hopkins University and the Medical University of South Carolina.

**Fig. 1:**
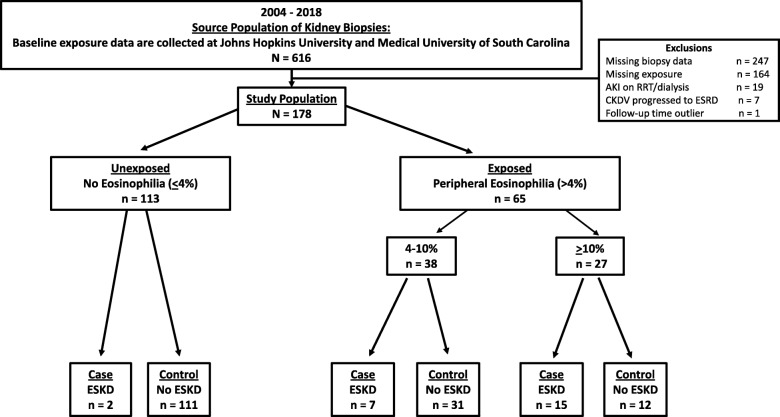
Selection of participants in a nested case-control study from a prospective kidney biopsy study

From the 616 eligible patients, 178 patients were identified through the registry of kidney biopsies excluding patients with kidney failure requiring dialysis (*n* = 19), dialysis-dependent chronic kidney disease (CKD) stage V patients with biopsy confirmed ESKD at the time of enrollment (*n* = 7), and those with missing biopsy or hematology data (*n* = 411) (Fig. 1). Patients were 18 years and older who underwent kidney biopsy for confirmation of their kidney diagnosis. We included patients who met the Kidney Disease Improving Global Outcomes (KDIGO) criteria for acute kidney injury (AKI) [12] with and without abnormal clinical findings of hematuria, pyuria or proteinuria. For each case, two controls were selected and matched on sex, age and duration of follow-up time since biopsy, so that 24 cases were matched to 48 controls. While we could not match on diagnoses given limited sample size, we did demonstrate the spectrum of etiologies in those with and without peripheral eosinophilia.

### Exposure measurement

Incident peripheral eosinophilia was assessed at enrollment using hematology reports at the time of kidney biopsy and analyzed as a binary variable. Eosinophil percentage was used instead of absolute eosinophil count for clinical relevance and as previously described [6], no eosinophilia as ≤4% of peripheral blood leukocytes (WBC) and peripheral eosinophilia as > 4% of WBC. We further categorized the severity of peripheral eosinophilia as 4–10%, and ≥ 10%.

### Outcome measurement

The primary outcome was defined as incident progression to ESKD, classified by estimated glomerular filtration rate (eGFR) ≤5 mL/min/1.73m^2^, an International Classification of Diseases Ninth/Tenth (ICD-9/10) revision code for a kidney disease-related hospitalization or death, per nephrologists’ diagnosis for patients requiring renal replacement therapy, and/or repeat kidney pathology suggesting extensive chronic, irreversible changes in the biopsy specimen.

### Covariates

All socio-demographical and clinical information were obtained at enrollment using Epic electronic medical records (EMR). Past medical history (hx) of atopic illness, filarial disease, asthma, and kidney transplantation were defined as binary variables. Similarly, history of hypertension (HTN), diabetes and medication use of proton pump inhibitor (PPI) were defined as binary variables. eGFR was obtained as patients’ “normal” eGFR, as measured by the CKD-Epi equation [13], prior to study entry and assessed by combination of previous medical records and laboratory chemistries. Other baseline variables measured as continuous variables at the time of enrollment included serum creatinine (Cr), serum Immunoglobulin E (IgE) levels, complements (C3, C4), and proteinuria.

The indication for kidney biopsy was characterized by four categories as per the nephrologists’ standard orders in ICD-9/10: AKI, CKD, AKI on CKD (AOCKD), or nephrotic syndrome. We characterized urine proteinuria based on the urine-protein-creatinine ratio (UPCR) and on urinalyses, as trace, + 1, + 2, + 3, or + 4 as reported by standard laboratory processing. Urinalyses was also assessed for the presence of pyuria, urine eosinophils and hematuria.

Tissue from kidney biopsy specimens was processed in the pathology departments using standard methods for light, immunofluorescence, and electron microscopy. The exact locations of the eosinophils were captured on the tissue specimens, and other inflammatory markers (e.g. lymphocytes and plasma cells) were documented using individual biopsy reports. The number of eosinophils were documented as per high-power field (hpf) and refers to number of eosinophils in the renal interstitium. For the purpose of this study, pathologists categorized kidney tissue eosinophils as: “rare” if < 5 per hpf, “few” if 5–10 per hpf, “many” if > 10 per hpf, and “numerous” if > 25 per hpf, as previously documented [14]. Pathologists independently evaluated biopsy slides to establish primary and secondary diagnoses, including acute tubular injury, chronic changes, or other kidney biopsy abnormalities such as IN.

### Statistical methods

Analysis of variance (ANOVA) and X^2^ t-test were used for statistical analysis on demographics and clinical characteristics. Results were reported as proportions for binary or categorical variables and mean for continuous variables. Pearson’s correlation was used to evaluate possible correlations amongst all the variables, but since no strong correlations existed, none of the variables were eliminated. Sex and age are known, strong confounders in ESKD and therefore matched upon [15]. Race was not matched in order to evaluate the independent effect of it on our outcome. Every case (*n* = 24) was matched to two controls, of the same sex, age and follow-up time (months) from biopsy.

Matched odds ratios (OR) for ESKD, calculated, as an estimate of the hazard ratio, and corresponding 95% confidence (CIs) were estimated using conditional logistic regression. Both univariate and multivariate models were used to show associations. A final multivariate model was created through stepwise elimination of variables of interest from univariate analysis while biologically relevant variables were retained, with the intent of using one variable for every 10 outcomes to avoid overfitting of the model. Additional analyses were conducted for baseline clinical demographics and statistical significance was determined with the use of likelihood-ratio test. UPCR, eGFR, HTN were included in multivariate models because they are strong predictors for ESKD [16, 17]. All analyses were performed using Stata version 15.1 (StataCorp, College Station, TX) [18].

Sensitivity analyses were performed using peripheral eosinophilia as a continuous variable. Univariate and multivariate analyses showed significant associations with higher degree of peripheral eosinophilia and ESKD. UPCR was also modeled as a binary variable and per KDIGO guidelines, normal UPCR defined as ≤0.5 mg/dl in 24-h urine [19]. The area under the ROC (AUC) was calculated to assess the ability of peripheral eosinophilia to discriminate between ESKD progressors and non-progressors.

## Results

In the overall study population, 65 (37%) of 178 patients had peripheral eosinophilia and 113 (63%) had no eosinophilia, 101 (57%) were male, 88 (49%) white and mean age of 52 ± 17 years. Among those with peripheral eosinophilia, 38 (58%) had 4–10% eosinophilia and 27 (42%) ≥10% eosinophilia. Those with peripheral eosinophilia were significantly males with higher baseline eGFR ≥60 ml/min/1.73m^2^ and higher mean Cr at enrollment, but without significant hx of HIV, kidney transplantation or asthma (Table 1). Overall, the most common reasons for nephrology consultation and kidney biopsy were AOCKD (40%), AKI (38%) and nephrotic syndrome (17%). Table 2 demonstrates the spectrum of etiologies which were comparatively balanced in this cohort when stratified by eosinophilia, however, there was a higher proportions of ANCA-associated vasculitis, FSGS, and lupus nephritis in the cohort of patients without eosinophilia compared to patients with peripheral eosinophilia, albeit non statistically significant.

**Table 1:** Baseline characteristics of patients who underwent kidney biopsy and their baseline eosinophilia on hematology

Demographics	No Eosinophilia ≤ 4% (*n* = 113)	Peripheral Eosinophilia > 4% (*n* = 65)	*P*-value
Female	56 (50%)	21 (32%)	0.025
Age, mean (SD), y	51 (17)	53 (18)	0.51
Race			
White	57 (50%)	31 (48%)	0.55
Black	47 (42%)	30 (46%)	
Other	9 (8%)	4 (6%)	
Asthma	17 (15%)	8 (12%)	0.61
Filarial Disease	0 (0%)	1 (2%)	0.19
HIV	15 (14%)	4 (6%)	0.13
Transplant	18 (16%)	8 (13%)	0.54
HTN	80 (71%)	42 (70%)	0.84
Baseline eGFR stage^a^			
I	63 (56)	49 (76)	0.013
II	35 (31)	9 (13)	
III	10 (8)	3 (5)	
IV	1 (1)	3 (5)	
early V	4 (4)	1 (1)	
Diabetes	19 (17)	15 (23)	0.32
Mean UPCR (SD), g	3.4 (9.9)	3.2 (4.5)	0.86
Mean serum Cr at biopsy (SD), mg/dl	2.9 (2.9)	3.9 (3.9)	0.043
Indication for kidney biopsy			
AKI	45 (40)	23 (37)	0.03
CKD	5 (4)	0 (0)	
AKOCKD	39 (27)	35 (53)	
Nephrotic Syndrome	24 (21)	7 (10)	

Results expressed as n (%), unless otherwise indicated

^a^based on eGFR by CKD-Epi equation

Abbreviations: *HIV* human immunodeficiency virus; *HTN* hypertension; *eGFR* estimated glomerular filtration rate; *UPCR* urine-protein-creatinine-ratio; *Cr* creatinine; *AKI* acute kidney injury; *CKD* chronic kidney disease; *AOCKD* acute on chronic kidney injury

**Table 2:** Etiology of kidney diseases stratified by presence or absence of eosinophilia

Primary Diagnosis	No Eosinophilia *n* = 113	Peripheral Eosinophilia *n* = 65
Interstitial nephritis	5 (4)	18 (28)
Diabetic nephropathy	14 (12)	9 (14)
Hypertensive arteriosclerosis	7 (6)	4 (6)
Primary focal segmental glomerulosclerosis	19 (17)	3 (5)
Membranous nephropathy	3 (3)	5 (8)
Membranoproliferative glomerulosclerosis	14 (12)	5 (8)
Lupus nephritis	10 (9)	2 (3)
ANCA vasculitis	11 (10)	5 (8)
Acute tubular injury	6 (5)	8 (12)
T cell/Antibody-mediated rejection	7 (6)	1 (2)
Amyloid	3 (3)	0
Thrombotic microangiopathy	4 (4)	1 (2)
Scleroderma	2 (2)	1 (2)
IgA nephropathy	1 (1)	1 (2)
Other^a^	7 (6)	2 (3)

Displayed as n (%)

ANCA defined as antineutrophil cytoplasmic antibody

^a^Other defined as minimal change disease, oxalate nephropathy, Alports disease, IgG Kappa nephropathy, calcineurin toxicity, thin basement membrane, post-infectious glomerulonephritis

Of those patients who progressed to ESKD and had peripheral eosinophilia (*n* = 22), biopsy confirmed primary clinical diagnoses of IN, diabetic nephropathy, acute tubular injury, arteriosclerosis, ANCA-associated vasculitis and primary focal segmental glomerulosclerosis (FSGS) (Table 3). Overall, median follow-up time to incident ESKD was 36 (interquartile range [IQR] 49) months. The median duration to ESKD was 32 (IQR 63) months, among 4–10% eosinophilia patients, and 36 (IQR 46) months, among ≥10% eosinophilia patients (*P =* 0.14) (Table 5). Those patients with peripheral eosinophilia had a statistically significant relationship with ESKD compared to those without eosinophilia (*P* <  0.001) (Table 5).

**Table 3:** Etiology of kidney diseases among cases, those who progressed to ESKD, stratified by presence or absence of eosinophilia

Primary Diagnosis	No Eosinophilia (*n* = 2)	Peripheral Eosinophilia (*n* = 22)
Interstitial nephritis	–	7 (31)
Diabetic nephropathy	–	4 (18)
Hypertensive arteriosclerosis	–	2 (9)
Primary focal segmental glomerulosclerosis	1 (50)	1 (5)
Membranous nephropathy	–	–
Membranoproliferative glomerulosclerosis	–	–
Lupus nephritis	–	–
ANCA vasculitis	–	2 (9)
Acute tubular injury	–	4 (18)
IgA nephropathy	–	1 (5)
T cell/Antibody-mediated rejection	1 (50)	1 (5)
Amyloid	–	–
Thrombotic microangiopathy	–	–
Scleroderma	–	–
^a^Other	–	–

Displayed as n (%)

ANCA defined as antineutrophil cytoplasmic antibody

^a^Other defined as minimal change disease, oxalate nephropathy, Alports disease, IgG Kappa nephropathy, calcineurin toxicity, thin basement membrane, post-infectious glomerulonephritis

**Fig. 2:**
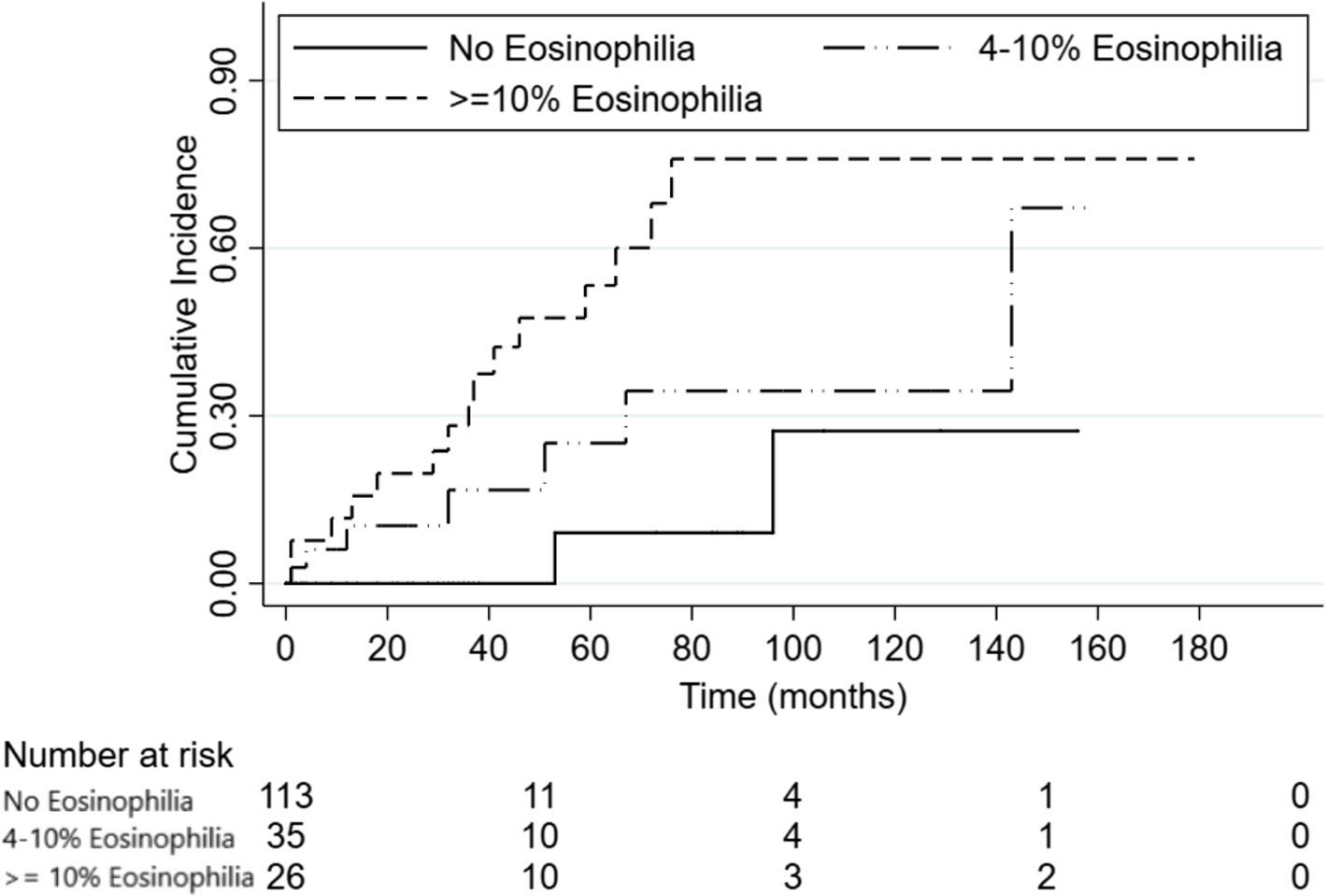
Progression to end-stage-kidney-disease by eosinophilia in the cohort study

Figure 2 depicts the fastest decline of kidney function among those with ≥10% eosinophilia compared to those with 4–10% eosinophilia or no eosinophilia. Half of those with eosinophilia ≥10% progressed to ESKD by approximately 60 months. After stratifying by baseline eGFR, majority of patients had higher stages of baseline eGFR in stages I-III with eGFR ≥30 ml/min/1.73 m2 (Table 4). Table 5 demonstrates a 4–10% peripheral eosinophilia rate was associated with 22 kidney tissue eosinophils per hpf (standard deviation [SD] 20) compared to those patients without eosinophilia that had 3 kidney tissue eosinophils per hpf (SD 7). Patients with ≥10% eosinophilia had 19 (SD 18) kidney tissue eosinophils per hpf. Tissue eosinophilia increased linearly for every 1% increase in peripheral eosinophilia (*P* <  0.001) (Table 5).

**Table 4:** Presence or absence of eosinophilia cases, who progressed to ESKD, and controls, who did not progress to ESKD, stratified by baseline kidney function

Baseline Kidney Function^a^	ESKD		No ESKD	
	No Eosinophilia (*n* = 2)	Peripheral Eosinophilia (*n* = 22)	No Eosinophilia (*n* = 111)	Peripheral Eosinophilia (*n* = 43)
Stage I	–	18 (81)	63 (57)	31 (74)
Stage II	1 (50)	1 (5)	34 (31)	8 (18)
Stage III	1 (50)	1 (5)	9 (8)	2 (4)
Stage IV	–	2 (9)	1 (1)	1 (2)
Early Stage V	–	–	4 (3)	1 (2)

Displayed as n (%)

^a^based on eGFR by CKD-Epi equation

**Table 5:** Progressors and non-progressors to ESKD stratified by the presence and absence of eosinophilia in the study population

	No Eosinophilia ≤ 4% (*n* = 113)	Peripheral Eosinophilia 4–10% (*n* = 38)	Peripheral Eosinophilia ≥10% (*n* = 27)	P-value*
ESKD, n (%)	2 (2)	7 (18)	15 (56)	< 0.001
No ESKD, n (%)	111 (98)	31 (82)	12 (44)	
Time to ESKD, median (IQR), months	74 (43)	32 (63)	36 (46)	0.14
Kidney tissue eosinophil				
n (%)	96 (74)	27 (71)	14 (52)	–
mean (SD), hpf	3 (7)	22 (20)	19 (18)	< 0.001**

*P-value calculated by analysis of variance (ANOVA) test of means for continuous variables and categorical variables. X^2^ test calculated for binary variables. *P* < 0.05 considered statistically significant

Abbreviations: *ESKD* end-stage-kidney-disease

Peripheral Eosinophilia is defined as eosinophils > 4% of blood leukocytes; Time to ESKD defined as months from the time of kidney biopsy

**linear association of peripheral eosinophilia on tissue eosinophils per high-power field (hpf)

Progressors to ESKD were more likely to have peripheral eosinophilia (92% cases versus 27% controls, *P* <  0.001) and have higher UPCR at the time of biopsy at 4.7 g/g (SD 5.4) in cases versus 2.4 g/g (SD 3.0) in controls (*P* < 0.039). History of asthma, HIV, kidney transplantation or filarial disease were not associated with ESKD. The presence of urinary eosinophils also had a positive, but non-significant association with ESKD in 72 patients (OR 6.4 [0.8, 53.9], *P* = 0.087) (data not shown).

**Fig. 3:**
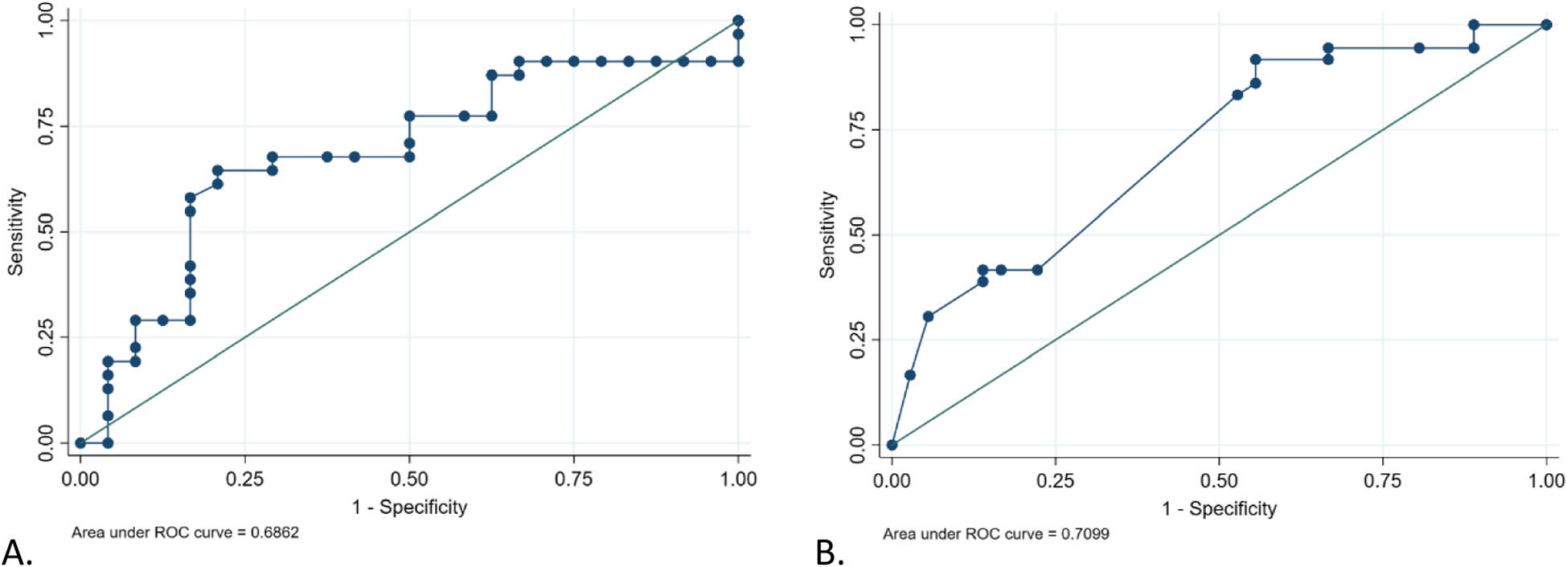
AUC of peripheral eosinophilia to predict progression to ESKD in this study population and sensitivity analysis. **a** The AUC of peripheral eosinophilia on predicting ESKD progression using continuous urine-proteincreatinine (UPCR) ratio (AUC 0.69). **b** Sensitivity analysis performed for AUC of patients with peripheral eosinophilia and the progression to ESKD using UPCR as a binary variable, cutoff <0.5mg/dl in 24-hour urine per KDIGO guidelines, (AUC 0.71) [12]

Presence of peripheral eosinophilia was associated with higher risk of progression to cases of ESKD (crude OR 6.7 [2.1, 21.1], *P* < 0.001) compared to those who did not progress to ESKD. In univariate model, there was 8-fold higher risk of progression to ESKD after adjusting for baseline eGFR (OR 8.2 [2.0, 33.0], *P* = 0.003). The association was also significantly increased after adjusting for HTN (OR 7.4 [2.4, 23.3]), race (OR 7.9 [2.4, 26.1]), or diabetes (OR 6.7 [2.1, 21.4]) in univariate models. Adjusting for baseline eGFR, UPCR and hypertension, patients with peripheral eosinophilia had approximately 15-fold higher association with ESKD (OR 15.9 [1.9, 134.7]) compared to those without eosinophilia. African Americans had a significant 3-fold higher risk of ESKD compared to whites (OR 3.4 [1.1, 9.9], *P* < 0.001), when adjusted for eosinophilia. In the overall study population, the AUCs for peripheral eosinophilia in predicting progression to ESKD during follow-up was 0.69 compared to AUC of 0.71 in sensitivity analysis, where UPCR was used as binary variable, according to KDIGO normal and abnormal values of UPCR (Fig. 3).

## Discussion

These prospective findings demonstrate a positive association between peripheral eosinophilia and the subsequent progression to ESKD with greater than 15-fold higher risk, even after fully adjusted models 15.9 [1.9, 134.7]). This association was more evident in African Americans when compared to Caucasians. Overall, in the study population, mean follow-up was 64 ± 49 months. These results suggest that the association between ESKD and peripheral eosinophilia is independent of HTN, UPCR, eGFR, age, sex, and may be independent of race, diabetes and Cr in larger sample sizes. Moreover, patients with peripheral eosinophilia who progressed to ESKD had a higher presence of tissue eosinophil infiltration on biopsy specimens.

Generally, eosinophilia is associated with allergies, parasitical infections, fungal infections, asthmatic conditions, and drug reactions (e.g. nonsteroidal anti-inflammatory drug [NSAID], antibiotics, PPI). There are a number of case reports that have highlighted an association of eosinophilia in kidney diseases such as kidney carcinoma [4, 20–22], thrombotic thrombocytopenic purpura [23], transplant rejection [24–26], and kidney replacement modalities [27–30]. However, eosinophilia has not been used as a marker for determining future risk of kidney disease. Patients with eosinophilia who developed ESKD in our cohort were less likely to have a history of asthma, filarial disease, atopic disease, allergic responses and were more likely to have diagnoses other than IN on kidney biopsy, such as diabetic nephropathy, acute tubular injury, hypertensive kidney disease and IgA nephropathy. This is consistent with a prior report highlighting the presence of urine eosinophils in other non-specific kidney pathologies other than IN [14, 31]. In our study, the presence of urinary eosinophils had a positive, but non-significant association with ESKD in 72 patients.

Physiological studies have shown eosinophils are activated by receptors in response to inflammatory and immunological pathways in the presence of an allergen or pathogen, resulting in the release of cytokines, chemokines and T cells [2, 32]. Eosinophils can express MHC class II and may act as antigen presenting cells. After traveling to regional lymph nodes, where they encounter CD4 T cells, eosinophils promote proliferation and cytokine production of IL-4, IL-5, and IL-13. A potential key component may be via upregulation of dendritic cells, mast cells, basophils, neutrophils and T cells [2]. One theory is that eosinophils are mediated by specific types of T-helper cells that result in higher cytokine production, including TNF-α and IL-9, and this contributes to interstitial atrophy, irreversible fibrosis, and eventually ESKD [33]. This theory is evident in recent studies in the field of IgG4-related kidney disease, where peripheral and tissue eosinophilia can progress to irreversible fibrotic dysfunction or organ failure [34, 35]. The upregulation of T-helper cells and cytokines IL-4, IL-5, IL-13, and IL-21, are thought to trigger a cascade of IgE production, macrophage activation and differentiation of B cells to plasma cells, whereby producing IgG4. Upregulation of T cells may also result in activation of TGF-β, increasing fibroblast activation and promoting additional fibrosis formation. Similarly, Macdonald et al. have shown that kidney allograft dysfunction and acute vascular rejection are associated when there is extensive eosinophil infiltration in kidney biopsies [36]. Taken together, these previous studies support the paradigm that peripheral eosinophilia infiltration and accumulation of their cytokines stimulate fibroblast proliferation and promote tissue destruction, specifically in the kidney. This theory is supported by our study, which demonstrates higher tissue eosinophil infiltration in renal biopsies among ESKD progressors. Peripheral eosinophilia on routine laboratory results of patients undergoing kidney biopsy may indicate increased risk from aberrant inflammatory states, including underlying comorbidity(es) and specific medication use. Peripheral eosinophilia may be an indicator for kidney damage and the severity of this marker may predict future irreversible interstitial damage, similar to the utility of CRP as a good clinical marker for inflammation and atherosclerosis [37, 38].

There are several limitations to our study including a small study population, which made multivariate modeling challenging in order to avoid overfitting the model as well as presence of unmeasured covariates (i.e. use of polypharmacy medications). Second, peripheral eosinophilia was measured at single point in time, and there may be some element of residual confounding on repeat testing. Prognosis of kidney outcomes may be dependent on the etiology of kidney disease and was not matched in this cohort due to the limited sample size and could result in confounding and selection bias. However, both progressors and non-progressors had a similar spectrum of kidney disease diagnoses other than IN (Tables 2 and 3), with higher incidence of ANCA-associated vasculitis, tubular disease, hypertensive arteriosclerosis and diabetic nephropathy in the cohort of patients with eosinophilia who subsequently progressed to ESKD (*P* = 0.29). Similarly, the spectrum of disease with the exposure and outcomes were similar when stratified by baseline eGFR, where the majority of patients had higher stages of baseline eGFR from I-III, eGFR ≥30 ml/min/1.73m^2^ (Table 4).

Several factors should be considered in the interpretation of our findings. We believe our study highlights new information that has not been evaluated between eosinophilia and ESKD, specifically the higher presence of peripheral eosinophilia and higher risk of progression to ESKD. We believe that matching one case to two controls by sex, age and follow-up time post kidney biopsy increased the power and precision over the course of a 14-year study. Conducting a nested case-control study in a prospective cohort rather than a traditional case-control study reduces several forms of selection biases. Specifically, cases and controls are drawn from the population in a fully enumerated cohort; controls are selected independently of the exposure (i.e. exposed and unexposed controls are the same fraction of the exposed and unexposed in the source populations), decreasing the possibility of selection bias that can often be in traditional case-control studies where the controls are chosen by exposure [39]. The “time” temporality component is important in this study design; until the disease/outcome emerges, the patient is eligible as a control for other matching cases and contributes time to the control cohort. Temporality then allows for exposure to happen first and then the outcome, to lessen the concern of reverse causation. Thus, a “nested” case-control study reduces several forms of selection biases and allows for temporal relationship between the exposure and occurrence of outcome during the time period. Moreover, using a matched study design allowed direct estimation of risk from odds and matching on strong confounders of ESKD allowed for the measure of association with less concern for bias. Participants were hospitalized from two tertiary referral centers at Johns Hopkins Hospital and the Medical University of South Carolina, representing diverse cultural populations in the northern and southern areas of the USA.

## Conclusions

We conclude that eosinophilia can be seen in various kidney conditions, and peripheral eosinophilia together with tissue eosinophil infiltration is significantly associated with progression to ESKD. Future prospective population-based studies should be conducted to determine the significance of biopsy tissue eosinophil infiltration and peripheral eosinophilia in the progression of ESKD, with a better emphasis on medication, comorbid illnesses, repeat biopsies and types of kidney disease diagnoses.

## Data Availability

The datasets used and/or analyzed during the current study are available from the corresponding author on reasonable request.

## Abbreviations

AKI: Acute Kidney Injury
ANCA: Antineutrophil cytoplasmic antibody
ANOVA: Analysis of variance
AOCKD: AKI on CKD
C3, C4: Complements
CKD: Chronic kidney disease
Cr: Serum creatinine
CRP: C-reactive Protein
eGFR: Estimated glomerular filtration rate
EGPA: Eosinophilic granulomatosis with polyangiitis
EMR: Electronic medical records
ESKD: End-stage-kidney-disease
GPA: Granulomatosis with polyangiitis
hpf: High power field
HTN: Hypertension
Hx: History
ICD-9/19: International Classification of Diseases Ninth/Tenth
IgE: Immunoglobulin E
IN: Interstitial nephritis
KDIGO: Kidney Disease Improving Global Outcomes
MHC: Major histocompatibility complex
MPA: Microscopic polyangiitis
NSAID: Nonsteroidal anti-inflammatory drug
OR: Odds ratio
PPI: Proton pump inhibitor
UPCR: Urine-protein-creatinine ratio
WBC: White blood leukocytes
